# Acetate Promotes a Differential Energy Metabolic Response in Human HCT 116 and COLO 205 Colon Cancer Cells Impacting Cancer Cell Growth and Invasiveness

**DOI:** 10.3389/fonc.2021.697408

**Published:** 2021-08-03

**Authors:** Sara Rodríguez-Enríquez, Diana Xochiquetzal Robledo-Cadena, Juan Carlos Gallardo-Pérez, Silvia Cecilia Pacheco-Velázquez, Citlali Vázquez, Emma Saavedra, Jorge Luis Vargas-Navarro, Betsy Alejandra Blanco-Carpintero, Álvaro Marín-Hernández, Ricardo Jasso-Chávez, Rusely Encalada, Luz Ruiz-Godoy, José Luis Aguilar-Ponce, Rafael Moreno-Sánchez

**Affiliations:** ^1^Departamento de Bioquímica, Instituto Nacional de Cardiología, México, Mexico; ^2^Banco de Tumores, Instituto Nacional de Cancerología, México, Mexico; ^3^Departamento de Medicina Interna, Instituto Nacional de Cancerología, México, Mexico

**Keywords:** acetate thiokinase, cancer biomarker, colon cancer, acetylation, oxidative phoshorylation

## Abstract

Under dysbiosis, a gut metabolic disorder, short-chain carboxylic acids (SCCAs) are secreted to the lumen, affecting colorectal cancer (CRC) development. Butyrate and propionate act as CRC growth inhibitors, but they might also serve as carbon source. In turn, the roles of acetate as metabolic fuel and protein acetylation promoter have not been clearly elucidated. To assess whether acetate favors CRC growth through active mitochondrial catabolism, a systematic study evaluating acetate thiokinase (AcK), energy metabolism, cell proliferation, and invasiveness was performed in two CRC cell lines incubated with physiological SCCAs concentrations. In COLO 205, acetate (+glucose) increased the cell density (50%), mitochondrial protein content (3–10 times), 2-OGDH acetylation, and oxidative phosphorylation (OxPhos) flux (36%), whereas glycolysis remained unchanged *vs.* glucose-cultured cells; the acetate-induced OxPhos activation correlated with a high AcK activity, content, and acetylation (1.5–6-fold). In contrast, acetate showed no effect on HCT116 cell growth, OxPhos, AcK activity, protein content, and acetylation. However, a substantial increment in the HIF-1α content, HIF-1α-glycolytic protein targets (1–2.3 times), and glycolytic flux (64%) was observed. Butyrate and propionate decreased the growth of both CRC cells by impairing OxPhos flux through mitophagy and mitochondrial fragmentation activation. It is described, for the first time, the role of acetate as metabolic fuel for ATP supply in CRC COLO 205 cells to sustain proliferation, aside from its well-known role as protein epigenetic regulator. The level of AcK determined in COLO 205 cells was similar to that found in human CRC biopsies, showing its potential role as metabolic marker.

## Introduction

It has been established that several bacteria found in the microbiome of colon cancer patients secrete bioactive metabolites (reactivated estrogens, short-chain carboxylic acids (SCCAs), amino acids, secondary bile acids) that may modulate colon cancer growth (1–4). In particular, SCCAs (acetate, propionate, butyrate) have been detected at high levels (50–346 µmol/g wet tissue) in both plasma and feces from CRC patients (5, 6), compared to samples from healthy persons.

The high SCCAs pool might serve primarily as metabolic fuel to be oxidized by CRC mitochondria to support CRC cell proliferation. However, studies analyzing the role of SCCAs in the energy metabolism of cancer cells are scarce, which may be related to the wide-spread belief that cancer cells have an impaired mitochondrial metabolism (7, 8).

It is well documented that butyrate and propionate (0.5–10 mM), but not acetate, strongly inhibit cancer cell growth in human colon (HT-29), prostate (PC3 and DU-145), lung (H1299 and H1703), and breast (MCF-7) adenocarcinomas (9–12). Cancer cell growth impairment has been associated to the strong inhibition of histone deacetylase activity induced by butyrate or propionate (13–15). However, the accelerated colon cancer cell growth is not completely subdued when histone deacetylase activity is suppressed by propionate or butyrate, indicating that cell growth also depends on other processes/factors not sensitive to propionate/butyrate.

On the other hand, acetate could be an important fuel for colon cancer mitochondria even in the presence of glucose or fatty acids. Indeed, it has been demonstrated that acetate oxidation may promote the Crabtree effect (16) by stimulating *de novo* lipid biosynthesis in breast cancer MDA-MB-468 cells (17), which may not favor OxPhos [reviewed in (18)]. In addition to its role as carbon source, acetate may also promote acetylation, a reversible post-translational covalent modification affecting histone and non-histone proteins. Key regulatory enzymes include histone deacetylases (HDACs) and histone acetyltransferases (HATs). In this regard, it has been documented that histone acetylation induced by acetate promotes lipid synthesis, cell migration, and cell adherence to extracellular matrix in cancer cells (19, 20). On the other hand, HATs have been implicated in the regulation of several proteins associated to signaling and metabolic pathways in non-cancer, cancer, and cancer stem cells (19, 21).

Therefore, the present study analyzes the effect of different SCCAs on cell growth, mitochondrial function, acetylation profile of energy metabolism proteins, and cell invasiveness, an ATP-dependent process. To this end, an integral analysis of the mitochondrial SCCAs oxidation was carried out in two widely used human CRC lines, COLO 205 and HCT 116 cells, by assessing the activity of acetate thiokinase (AcK), the initial step in the acetate oxidation pathway, the level of several OxPhos enzymes and their acetylation profile, and the OxPhos flux. To extend our findings to a more physiological setting with potential clinical relevance, analysis of AcK protein content and acetylation was also performed in human CRC biopsies. Our results clearly indicate that acetate (but not butyrate or propionate) at physiological doses was actively oxidized by COLO 205 cells, through a highly over-expressed and very active AcK, to sustain cancer growth. In contrast, HCT 116 cells showed a low AcK activity and decreased OxPhos capacity, which was compensated by glycolysis activation. In both CRC cell lines, butyrate and propionate decreased mitochondrial function, leading to ROS accumulation and cellular death by autophagy and mitochondrial fragmentation.

## Material and Methods

### CRC Cell Growth

Human metastatic colorectal COLO 205 and HCT 116 cells and triple negative breast cancer MDA-MB-231 cells were grown in Dulbecco’s Modified Eagle Medium (DMEM) supplemented with 10% fetal bovine serum (GIBCO; Rockville, MD, USA) *plus* 10,000 U penicillin/streptomycin (Sigma; Steinheim, Germany) and incubated under 5% CO2, 95% air at 37°C until 80–90% confluence. COLO 205 and HCT 116 cell genotyping analyses, performed by the National Institute of Genomic Medicine (INMEGEN, México), revealed that they shared 7 out of 15 and 16 out of 18 alleles, respectively, reported by the ATCC for their authentication. For cellular growth in the presence of the different SCCAs, COLO 205 and HCT 116 cells at 10,000 cells/0.1 ml were cultured in 96 multi-well plates in DMEM medium *plus* 5 mM glucose for 6 days. Acetate, propionate, or butyrate was added at the beginning of the cultivation (day 0) at 5 mM (acetate) or 0.1 mM (propionate or butyrate) final concentration. Acetate was also added to the culture medium in combination with butyrate at the doses previously indicated.

### Human CRC Biopsies

As a merely exploratory translational study in human biopsies, the number of samples used was small, as has been published for other similar studies (22–24). Five colorectal carcinoma samples were collected at the Instituto Nacional de Cancerología, México, as described before (24), following the handling protocols approved by the Committees of Ethics and Research of Instituto Nacional de Cancerología, México (INCAN) (http://incan-mexico.org/incan//pub/investigacion/bioetica/Anexo7.pdf), and supported by patients’ informed consents according to the Declaration of Helsinki. The colorectal tissue from at least five hepatoma AS-30D-containing Wistar rats was used as positive control, whereas colorectal samples from non-cancer Wistar rats were used as negative control. All human biopsy samples and rat tissues were stored in liquid nitrogen until their use as previously described (24).

### Western Blot Assays

Cells (4 × 10**^6^** cells/ml) were cultured in 60 × 15 cm Petri culture dishes. After each SCCA treatment, cells were lysed in RIPA (50 mM Tris pH 7.4, 1% Nonidet-P40, 0.25% sodium deoxycholate, 150 mM NaCl, 1 mM EDTA, 1 mM PMSF, 1 mM sodium fluoride, and 1 tablet of protease inhibitors cocktail) buffer and collected. Protein preparations (50 μg) were separated by SDS‐PAGE and transferred to polyvinylidene fluoride (PVDF) membranes (BioRad; Hercules, CA, USA). Western blot analysis was performed by immunoblotting with anti-HIF1α, -GLUT-1, -GLUT-3, -HKI, -HKII, -HPI, -2-OGDH, -ATPS, -ND1, -ANT, -P-AMPK, -Beclin, -DRAM, -LAMP1, -PGC-1α, -PINK-1, -PARK-1, -FIS-1, -MFN-2, and -α-tubulin (1:1,000) or anti-AcK, -K-RAS, -SNAIL, -E-cadherin, -vimentin, -fibronectin (1:500) (Abcam; Cambridge, MA, USA) antibodies for total extracts. The hybridization bands were revealed with the corresponding secondary antibodies conjugated with horseradish peroxidase (Santa Cruz; CA, USA) and the ECL-plus detection system (Amersham; Buckinghamshire, UK). Percentage of each enzyme represented the mean ± S.D. of at least three independent experiments.

Human biopsy samples were resuspended and homogenized with a Teflon-pestle in 25 mM Tris–HCl buffer, 1 mM PMSF, 1 mM EDTA, and 5 mM DTT and centrifuged at 10,000 rpm for 30 min at 4°C (24). Protein samples (50 μg) were resuspended in loading buffer with 10% glycerol, 2% SDS, and 5% β-mercaptoethanol and subsequently separated by SDS-PAGE in 10 or 12.5% polyacrylamide gels. Afterwards, proteins were blotted onto PVDF membranes and incubated overnight with the following antibodies: anti-AcK, -K-RAS, and -α-tubulin (Santa Cruz Biotechnology; Santa Cruz, CA, USA) at 1:1,000 dilution. Bands of hybridization were detected with the corresponding secondary antibodies and the horseradish peroxidase reaction as previously described (25). Densitometry analysis was carried out using the Scion Image software (Scion; Walkersville, MD, USA). Normalization of all samples was performed against its respective loading control (α-tubulin), which was considered as 100% (24).

### Immunoprecipitation Assays

To assess the AcK, GLUT-1, GLUT-3, HKI, HKII, HPI, ND1, 2-OGDH, ANT, and ATPS, acetylation status, all these proteins were immunoprecipitated with their respective specific antibodies or with IgG1 (1 μg) for 1 h *plus* protein A (Sigma-Aldrich, St. Louis, MO, USA) at 4°C. Acetylation status was detected in the immunoprecipitated proteins with anti-acetyl-Lysine antibodies (1:1,000 dilution; Abcam) following manufacturer instructions (26).

### Acetate Thiokinase (AcK) Activity

HCT 116 and COLO 205 cytosol-enriched fractions were prepared from cells incubated in the absence or presence of the different SCCAs for 5 days. The cells were trypsinized, collected in Tris–HCl buffer (25 mM Tris–HCl, 1 mM EDTA. 5 mM DTT, 1 mM PMSF), and disrupted by freezing-thawing thrice. The homogenate was centrifuged at 3,500 rpm for 5 min at 4°C (27). AcK activity (EC 6.2.1.1; acetate-CoA ligase; acetate + CoA + ATP → acetyl-CoA + AMP + PPi) was determined at 37°C using a coupled enzymatic assay with pyruvate phosphate dikinase (PPDK; PEP + AMP + PPi → Pyr + ATP) from *E. histolytica* (28) and commercial lactate dehydrogenase (LDH). The incubation buffer contained 25 mM MOPS adjusted to pH 7.2. The reaction contained 10 mM acetate, 0.2 mM NADH, 10 mM CoA, 2 mM ATP, 10 mM MgCl2, 2 mM PEP, ~1U PPDK, 1.1 U LDH (Sigma, St. Louis, MO, USA) and 200–600 μg of cell protein. The addition of 0.02% Triton X-100 did not increase AcK activity, but it rather slightly inhibited it. NADH oxidation was monitored spectrophotometrically at 340 nm after specifically starting the reaction by adding acetate. Controls were done to ensure that the reaction rate was (i) under conditions of initial velocity (saturation of substrates, linear dependence on cell protein concentration, excess of coupling enzymes); and (ii) specific (reaction was started by adding one specific substrate-acetate; no activity was attained in the absence of any of the specific substrates; spurious activity was always subtracted); formate, propionate, or butyrate at 10 mM was unable to trigger the AcK reaction, indicating high specificity for acetate. The stock solutions of all substrates were always calibrated before use (28).

### Energy Metabolism Pathway Fluxes

For glycolysis flux, intact CRC cells (2 mg protein/ml) were incubated in 1.5 ml Krebs-Ringer medium as previously described (29). Briefly, aerobic glycolysis was carried out in an orbital shaking water bath with cells incubated in plastic flasks at 37°C. The reaction mixture contained cancer cells incubated in Krebs Ringer medium in a final volume of 3 ml. The reaction was started by the addition of exogenous glucose (5 mM). At time 0 and 10 min, the reaction was stopped by mixing a cell aliquot with 3% (w/v) cold perchloric acid and centrifuged. In parallel sets of experiments, cells were also incubated for 0 and 10 min with 2-deoxyglucose (2-DG, 10 mM) to correct for lactate production by glutaminolysis. Residual lactate production (2-DG insensitive lactate formation) was totally blocked by 5 µM rotenone (inhibitor of respiratory chain site 1).

The supernatants were neutralized with 1 N KOH/100 mM Tris. Lactate was enzymatically determined by using LDH (Roche, Mannheim, Germany) following the NADH formation at 340 nm (30). The contribution of glycolysis to the cellular ATP supply was determined from lactate production, assuming a stoichiometry of 1 mol of ATP produced *per* 1 mol of lactate produced.

For OxPhos flux, cancer cells (4 mg protein/ml) were incubated at 37°C in 1.9 ml of air-saturated Krebs-Ringer medium *plus* 5 mM glucose, and the rate of 5 µM oligomycin sensitive-O2 consumption was determined by using a high-resolution Oxygraph-2k (O2k, OROBOROS Instruments, Innsbruck, Austria) (31). The contribution of OxPhos to the cellular ATP supply was determined from the oligomycin-sensitive respiration rate multiplied by the ATP/O ratio that corresponds to 2.5 in cancer mitochondria respiring on NADH-linked substrates (32). This ratio value was used because it is indeed similar to that determined by other researchers using non-cancer mitochondria (33, 34) and represents the amount of ATP synthesized (by the ATP synthase) per oxygen consumed (by the cytochrome c oxidase in the respiratory chain); the units of the ATP/O ratio would be nmol ATP/ng atom oxygen.

### Isolation of AS-30D Hepatoma and Liver Mitochondria

Mitochondria were isolated from AS-30D hepatoma cells (35) or rat liver (36) using the standard sequential centrifugation method previously described. The mitochondrial protein content was determined by the Biuret assay using bovine serum albumin as standard. The mitochondrial and OxPhos functionality was evaluated in isolated mitochondria by measuring the rates of respiration in pseudo-state 4 (*i.e*., oxygen consumption by mitochondria incubated with Pi and oxidizable substrate before adding ADP), state 3 (*i.e*., oxygen consumption by mitochondria incubated with Pi and oxidizable substrate after adding exogenous ADP), and state 4 (*i.e*., oxygen consumption by mitochondria incubated with Pi and oxidizable substrate with ADP exhausted); and the coupling grade (i.e., respiratory control, RC = rate of state 3 respiration/rate of state 4 respiration) of both AS-30D and liver mitochondria. A high quality of mitochondrial preparations is revealed by RC values equal or greater than 4 (37–39).

Respiration of isolated mitochondria (1 mg protein/ml) was determined by polarography using a Clark-type O2 electrode in KME (120 mM KCl, 20 mM Mops, 1 mM EGTA) buffer pH 7.2 *plus* 2 mM KH2PO4 and the indicated oxidizable substrates.

For state 3 respiration 300–600 nmol ADP were added after 10–15 s incubation. Mitochondrial preparations with RC ratios greater than 4 in the presence of glutamate/malate as oxidizable substrate were commonly used for experimentation.

The transmembrane electrical potential (ΔΨm) of isolated mitochondria (0.5 mg/ml) was assayed in state 3 and pseudo-state 4 conditions with rhodamine 123 (50 nM). The changes in ΔΨm were monitored with a Shimadzu spectrofluorophotometer (RF-5301PC; Tokyo, Japan) at λexcitation = 488 nm and λemission = 575 nm. The maximal ΔΨm value was determined by measuring the change in the fluorescence signal before and after dissipation with the protonophore CCCP (1 µM) (40).

The content of mitochondrial citrate was determined as follows. Mitochondria (10 mg protein/ml) were incubated for 10 min in KME buffer + 2 mM K-phosphate at 37°C under smooth orbital shaking with glutamate (10 mM)/malate (5 mM) or acetate (5 mM)/malate (5 mM). After 10 min, 5 mM ADP was added; 2 min later, aliquots were withdrawn, mixed with ice-cold 3% (V/V) perchloric acid in 1 mM EDTA, and kept on ice. The aliquots were neutralized with 3M KOH/0.1 M Tris and stored at −72°C until use. Citrate was determined by a standard enzymatic assay (30).

### Mitophagy Assay

COLO 205 and HCT 116 cells (3 × 10^4^/ml) were cultured in 35-mm glass-bottomed Petri dishes (MatTek; Ashland, MA, USA) in the presence of glucose and the different SCCAs. To reveal mitochondrial subcellular localization, cells were washed and pre-incubated with MitoTracker Green (MTG, 0.5 µM). To reveal lysosomes, autophagosomes, and autolysosomes, cells were pre-incubated with LysoTracker Red (LTR, 0.5 µM). Cells were loaded with both dyes for 20 min at 30°C in DMEM. Time series of confocal images were collected every 1 to 2 min with a Zeiss LSM 510 meta inverted laser scanning confocal microscope (Carl Zeiss; Oberkochen, Germany) using 63X oil 1.4 N.A. plan apochromat objective lens. LTR λ_excitation_ of 543 nm was provided by a helium/neon laser, and λ_emission_ of 560 nm was measured. MTG λ_excitation_ of 488 nm was provided by an argon laser, and λ_emission_ of 500–550 nm was followed. Laser excitation energy was attenuated 1,000-fold to minimize photobleaching and photodamage (41).

### ROS Determination

Cells (1 × 10^4^ cells/ml) were cultured for 5 days in 96-well plates in DMEM in the presence of glucose and different SCCAs. After washing, cells were incubated with fresh free-serum DMEM (with no glucose or SCCAs), 25 μM dihydroethidium (DHE) was added, and ROS production was monitored every 30 s for 1 h with a Varioskan microplate reader (Thermo Fisher Scientific; Waltham, MA, USA) using λemission of 605 nm and λexcitation of 518 nm.

### Invasiveness Assays

COLO 205 or HCT 116 cells were incubated in free-serum DMEM with 5 mM glucose and different SCCAs. After 24 h, the cells were washed and resuspended again in fresh free-serum DMEM containing the respective SCCAs and placed in the upper compartment of 96-multiwell Boyden chambers (Merck Millipore; MA, USA) at a final concentration of 5 × 10^4^ cells/well for another 24 h at 37°C. The Boyden chamber lower compartment was filled with free-serum DMEM. The migrating cells, collected in the lower compartment, were identified with 60 nM calcein-AM after 60 min incubation for dye loading. Calcein fluorescence was detected at λexcitation of 485 nm and λemission of 520 nm by using a Varioskan microplate reader.

### Data Analysis

Experiments were performed with at least three independent samples. Data shown represent mean ± standard deviation (S.D.). For the detection of significant differences among several experimental groups, one-way ANOVA/*post hoc* Scheffé analysis was used (42).

## Results

### Effect of SCCAs on COLO 205 and HCT 116 Cellular Growth

**Figure 1:**
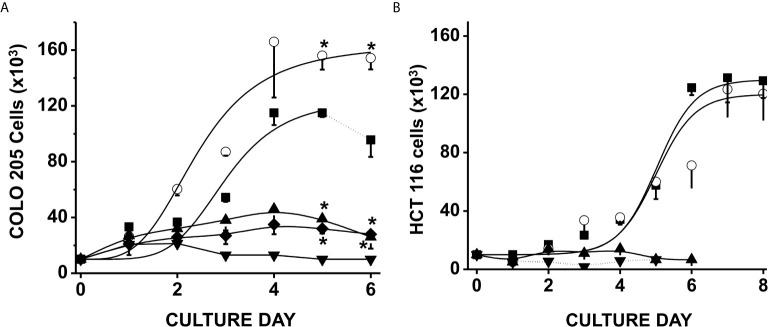
Effect of SCCAs on the growth of colorectal cancer cells. COLO 205 cells **(A)** and HCT 116 cells **(B)** were grown in the presence of 5 mM glucose alone (▪), or + 5 mM acetate (○), or 0.1 mM butyrate (▼) or 0.1 mM propionate (▲). Additionally, COLO 205 cells were grown in the presence of a mixture of 5 mM glucose + 5 mM acetate + 0.1 mM butyrate (♦). Solid lines represent the fitting to the experimental points by the exponential growth equation, which was made by using the Origin 8 software (Northampton, MA, USA). Dotted line at the end of 5 mM glucose condition indicates cellular death. Data shown represent the mean ± S.D. @ of at least three independent assays. *P < 0.05 *vs.* glucose alone.

Exposure of CRC cells to physiological concentrations of glucose (5 mM) + acetate (5 mM) significantly increased COLO 205 cell density by around 50% after 4–6 days of culture *vs.* control cells cultured with only glucose (**Figure 1A**); acetate also decreased the duplication time by 30% (from 59 ± 4 to 41 ± 5 h). In contrast, HCT 116 cell growth was unaffected by acetate + glucose (**Figure 1B**). Propionate or butyrate at 5 mM final concentration (+ 5 mM glucose) promoted a high cellular death (>95%) within the first days of cultivation (data not shown). In consequence, cells were exposed to much lower (0.1 mM) propionate or butyrate concentrations, at which both COLO 205 and HCT 116 cell growth was arrested, but viability was higher than 70% (**Figure 1**).

In order to assess a more physiological condition, a mixture of 5 mM acetate, 0.1 mM butyrate, and 5 mM glucose was added to COLO 205 cells, and its effect on cell growth was determined (**Figure 1A**). This SCCAs combination restrained CRC cell growth as it has been documented for RKO colorectal cells and THP-1 monocytic cells (43, 44), but cellular viability was not affected (>95%), indicating that acetate was not able to overcome butyrate-induced proliferation arrest. It is also worth noting that the CRC proliferation was abolished when the cells were cultured in the presence of any SCCA but with no glucose (data not shown), indicating the role of glucose as a carbon source to maintain CRC proliferation.

### Effect of SCCAs on CRC Cellular Energy Metabolism

Exposure of COLO 205 or HCT 116 cells to acetate, propionate, or butyrate in the presence of glucose for 5 days increased the levels of HIF-1α by 1.4 to 15.8 times *vs*. control cells (**Supplementary Figure 1**). In contrast, a significant decrease in the level of the glycolytic regulator P-AMPK was detected in (a) both cells cultured with acetate (24–40%); (b) both cells cultured with propionate (65–>90%); and (c) HCT 116 cells cultured with butyrate (60%). No changes in P-AMPK content were observed in COLO 205 cells exposed to butyrate (**Supplementary Figure 1A**). The increment in HIF-1α level positively correlated with a large increase in several flux-controlling glycolytic (GLUT-1, GLUT-3, and HKI) proteins (45) in both CRC cells exposed to the different SCCAs *vs.* cells exposed to only glucose. HKII remained unchanged in both colon cancer cells (**Supplementary Figures 1A, B** **)**, except for a significant increase (3.9 times) induced by butyrate in COLO 205 cells (**Supplementary Figure 1A**).

In parallel, levels of some OxPhos (ND1 and ANT) proteins also augmented by 2.6 to 9.9 times in both cell lines under acetate, propionate, or butyrate treatment. ATP synthase (ATPS) also increased in COLO 205 cells by 3.1–6.9 times (**Supplementary Figure 1A**), whereas it remained unchanged in HCT 116 cells (**Supplementary Figure 1B**).

**Figure 2:**
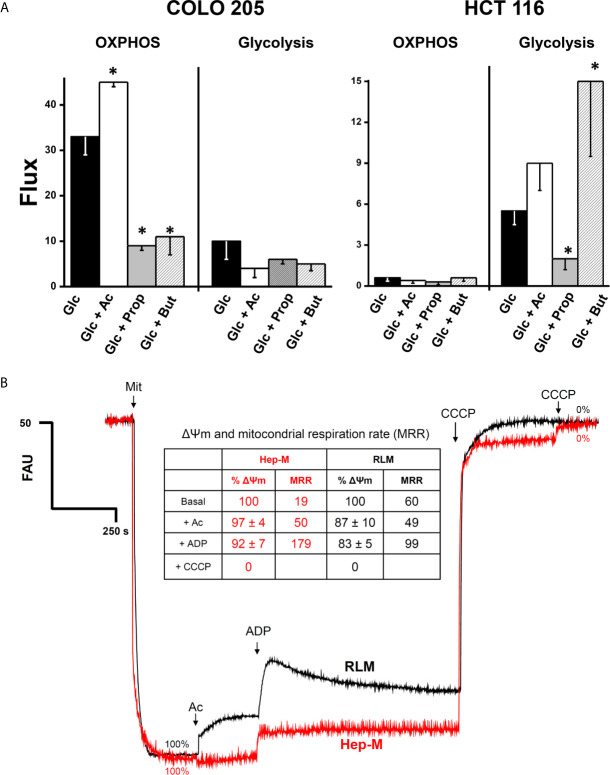
**(A)** Effect of SCCAs on the energy metabolism fluxes in COLO 205 and HCT 116 cells. Cells were cultured with the indicated carbon sources (5 mM glucose-Glc, 5 mM acetate-Ac, 0.1 mM propionate-Prop, 0.1 mM butyrate-But) for 5 days, harvested, washed, and used for energy fluxes determination. For OxPhos, flux represents the oligomycin-sensitive oxygen consumption expressed in ngAtO (nanogram atoms oxygen)/min/mg protein. For glycolysis, flux represents the 2DG-sensitive lactate production expressed in nmol of lactate produced/min/mg protein. The data shown are the mean ± S.D. @ of at least three different preparations. *P < 0.05 *vs.* Glc. **(B)** Effect of acetate on mitochondrial transmembrane potential (ΔΨm) in AS-30D hepatoma (Hep-M) and rat liver (RLM). Mitochondria (0.5 mg/ml) were incubated in KME buffer with malate (0.1 mM for Hep-M or (5 mM for RLM), 2 mM Pi, and 50 nM rhodamine 123. The experiment was initiated by the addition of mitochondria to the reaction mix. At the indicated times, acetate (Ac, 5 mM), ADP (300–600 nmol), and CCCP (1 µM final concentration) were added. Data in figure and inserted table represent the ΔΨm value in percentage calculated from **Supplementary Table 2**. Values shown between parentheses in bold letters, inside the inserted table, represent the mitochondrial respiratory rates expressed in ng atoms oxygen/min/mg protein taken from **Table 1** (Hep-M) and **Supplementary Table 1** (RLM).

The higher OxPhos protein contents found in COLO 205 cells exposed to acetate correlated with a significant 36% increment in the OxPhos flux (i.e., the oligomycin-sensitive oxygen consumption) (**Figure 2A**). In contrast, propionate and butyrate induced a strong decrease in COLO 205 OxPhos by 66–72%, despite the high ND1, ANT, and ATPS levels detected. In COLO 205 cells, acetate, propionate, or butyrate did not significantly modify the glycolysis flux (i.e., the total produced lactate sensitive to the glycolytic inhibitor 2-deoxyglucose) (**Figure 2A**). Nevertheless, OxPhos was the main ATP supplier (79–96%) in COLO 205 cells under all conditions. In contrast, HCT 116 cells showed impaired OxPhos in the presence of glucose alone or with the supplementation of SCCAs (**Figure 2A**), and it was markedly lower (<95%) than that of COLO 205 cells. To compensate for OxPhos impairment, HCT 116 cells exhibited an enhanced glycolysis rate, which provided much of the ATP (73–91%) required for cellular processes. Because of the scarce amount of biomass generated by COLO 205 and HCT116 cells cultured in the presence of propionate+glucose, butyrate+glucose, or acetate+butyrate+glucose, OxPhos was not determined in these cells.

In order to examine whether exogenous acetate promotes acetylation of energy metabolism proteins, which may impact the OxPhos functionality, the degree of (Lys) acetylation of several OxPhos as well as glycolytic proteins was determined in COLO 205 (**Supplementary Figure 2A**) and HCT 116 (**Supplementary Figure 2B**) cells. For COLO 205 cells cultured with acetate+glucose, significant higher acetylation of all proteins examined was attained (**Supplementary Figure 2A**), particularly of the mitochondrial 2-OGDH and glycolytic GLUT-1, but with the exception of GLUT-3, which showed less acetylation than in cells exposed to only glucose. For HCT 116 cells, acetate induced higher acetylation on ND1 and HK (I and II) isoforms, whereas low acetylation was observed in 2-OGDH, ATPS, and HPI (**Supplementary Figure 2B**).

### Effect of SCCAs on OxPhos, Mitochondrial Membrane Potential (Δψ_m_), and Intramitochondrial Citrate Content in Isolated Hepatoma AS-30D and Liver Mitochondria

It is certainly more rigorous to compare behavior and responses of CRC cells with their own isolated mitochondria. However, to obtain an enriched mitochondrial fraction of high quality and purity requires a large amount of cultured cancer cells. To overcome this situation, the rat AS-30D hepatoma model was used to grow a large cellular mass for production of high-quality mitochondrial preparations (23, 46). Therefore, in order to elucidate whether acetate, propionate, or butyrate may directly drive OxPhos (*i.e*., mitochondrial ATP synthesis), by serving as oxidizable substrates, and sustain other energy-dependent mitochondrial functions, we determined respiration rates (**Table 1**), Δψ_m_ (**Figure 2B** and **Supplementary Table 2**) and intramitochondrial citrate content (**Supplementary Table 3**) in mitochondria isolated from rat AS-30D hepatoma (Hep-M).

**Table 1:** SCCAs oxidation in mitochondria isolated from AS-30D hepatoma (Hep-M) cells.

Hep-M	Pseudo state 4 respiration (no ADP added)	State 3 (ADP-stimulated) respiration	State 4 respiration (after ADP exhaustion)	Respiratory control ratio
No added substrate	19 ± 10	19 ± 10	19 ± 10	1
0.1 mM Mal	57 ± 17	159 ± 80	64 ± 26	2 ± 1
0.5 mM Mal	74 ± 7	294 ± 44	74 ± 25	4 ± 1
1 mM Pyr + Mal	69 ± 19	349 ± 133	96 ± 29	4 ± 1
5 mM Pyr + Mal	74 ± 7	294 ± 44	74 ± 25	4 ± 1
5 mM Glu + Mal	36 ± 6	197 ± 64	41 ± 15	5.3 ± 2.3
Ac (mM) + Mal				
1	34 ± 8	175 ± 12	55 ± 5	3.1 ± 0.1
5	50 ± 4.5	179 ± 20	79 ± 20	2.3 ± 0.4
10	48 ± 8	189 ± 7	139 ± 17	1.3 ± 0.1
Prop (mM) + Mal				
1	45 ± 13	142 ± 60	142 ± 60	1
5	48 ± 15	115 ± 41	115 ± 41	1
10	47 ± 11	105 ± 37	105 ± 37	1
But (mM) + Mal				
1	45 ± 13	142 ± 60	142 ± 60	1
5	48 ± 15	115 ± 41	115 ± 41	1
10	47 ± 11	105 ± 37	105 ± 37	1

In all conditions, malate (Mal) was used at 0.1 mM; n = 3.

For comparative purposes and as a control, bioenergetic parameters were also evaluated in isolated mitochondria from rat liver (RLM), which is the tissue of origin of AS-30D hepatoma (**Supplementary Table 1** and **Figure 2B**). Because malate (≥0.5 mM) is rapidly oxidized by an active tumor mitochondrial matrix NADP^+^-dependent malic enzyme (23, 47), which is negligible in rat liver mitochondria (RLM), Hep-M was incubated with a lower malate concentration (0.1 mM). The rate of ADP-stimulated (state 3) respiration (i.e., oxygen consumption associated to OxPhos) and net state 3 respiration (state 3 respiration *minus* pseudo-state 4 respiration; as an index of OxPhos rate) in Hep-M with 0.1 mM malate was not stimulated by ADP (**Table 1**). Similarly, in the absence of 0.1 mM malate but in the presence of other exogenous NAD^+^-dependent substrates, ADP was unable to stimulate state 3 respiration rates (data not shown).

State 3 respiration in Hep-M incubated with acetate/malate was lower (2 times) than the net state 3 values obtained with pyruvate, the substrate derived from glycolytic glucose breakdown, suggesting that PDH is more efficient than AcK to generate mitochondrial acetyl CoA to drive ATP synthesis. However, other well-oxidized tumor mitochondrial substrates such as glutamate/malate maintained similar state 3 rates to those attained with acetate/malate (**Table 1**) (23, 48).

Propionate/malate or butyrate/malate caused either an apparent uncoupling or stimulated ATP hydrolysis, because state 4 respiration was enhanced and/or state 3 respiration remained elevated, unable to exhaust added ADP, resulting in a low respiratory control (**Table 1**). None of the substrates assayed affected the basal rate of respiration (*i.e*., pseudo-state 4 respiration).

In contrast to what was observed in Hep-M, ADP was unable to stimulate acetate-driven state 3 respiration rates in RLM (**Supplementary Table 1**) as has been reported (49). As expected, pyruvate/malate and glutamate/malate were oxidized at high rate by RLM (**Supplementary Table 1**). Propionate or butyrate inhibited state 3 respiration in RLM, whereas state 4 reached similar values as those with acetate/malate.

Acetate/malate oxidation in Hep-M and RLM was also monitored, measuring the Δψ_m_ under both state 3 (ADP-stimulated) and pseudo-state 4 (no ADP added) conditions (**Figure 2B** and **Supplementary Table 2**), as well as by assessing the content of intramitochondrial citrate (**Supplementary Table 3**). In Hep-M, acetate was able to generate and sustain a high Δψ_m_, correlating with the stimulated net state 3 respiration induced by acetate **(** **Figure 2B** **)**. In contrast, acetate addition induced in RLM a lower Δψ_m_ (**Figure 2B**), *i.e.*, a slight uncoupling effect, which was not observed in Hep-M, and lower net state 3 respiration. Similar Δψ_m_ values were found in Hep-M and RLM in both states 3 and 4 respirations supported by pyruvate, glutamate, or butyrate. Acetate/malate oxidation increased the content of citrate by 38% in Hep-M, indicating that acetate is actively oxidized as occurs in RLM to stimulate the Krebs cycle for NADH production (**Supplementary Table 3**).

### Acetate Thiokinase (AcK) Content, Acetylation Profile and Activity in CRC Cells and Human Colon Carcinoma Biopsies

**Figure 3:**
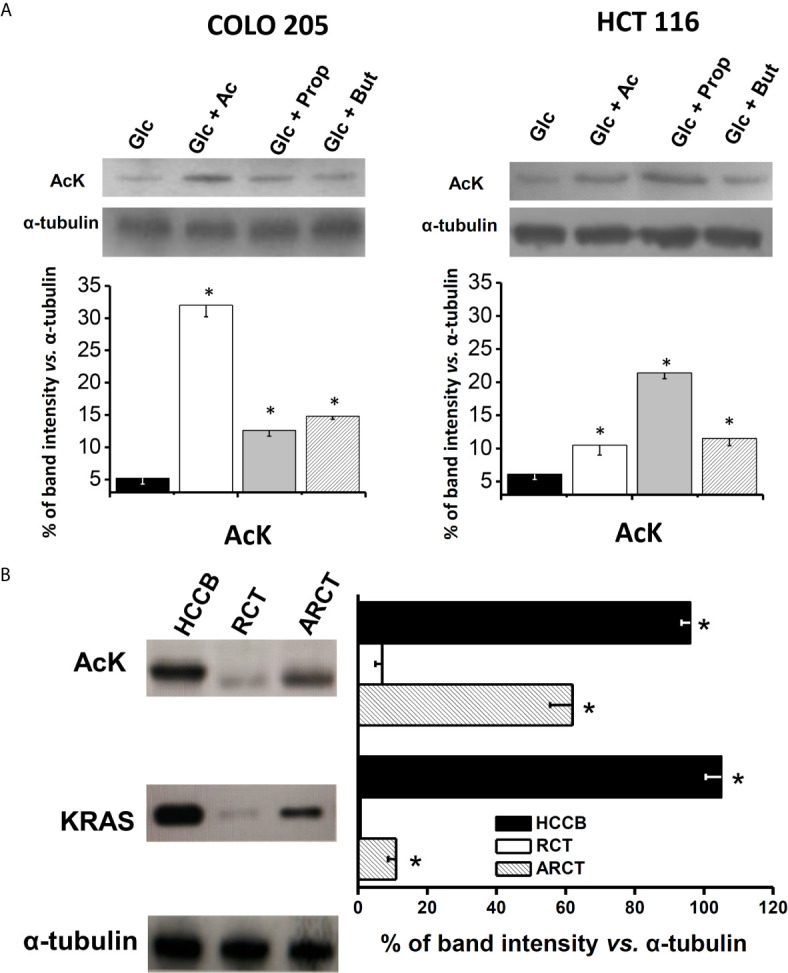
AcK protein content in **(A)** human colorectal cancer cells and **(B)** human cancer biopsy. The data shown represent the mean ± S.D. @ of at least three different preparations. *P < 0.01 *vs*. Glc for **(A)**; * P < 0.01 *vs*. rat colon tissue (RCT). HCCB, human colon cancer biopsy; ARCT, ascites rat colon tissue.

In order to better understand the relationship between acetate effects and its oxidation, the protein levels, acetylation degree, and activity of acetate thiokinase (AcK) were determined in both CRC cells. The AcK protein level (six times; **Figure 3A**), the degree of (Lys) acetylation (50%, **Supplementary Figure 3A**), and activity (3.2 times; **Table 2**) were significantly higher in COLO 205 cells exposed to acetate (+ glucose) *vs.* cells exposed to glucose alone. The slightly higher Ack (Lys) acetylation in COLO 205 cells exposed to acetate+glucose (**Supplementary Figure 3A**) correlated with an increase in enzyme activity, suggesting that this covalent modification is required for AcK activation. Although the AcK protein level was also higher in COLO 205 cells cultured with propionate or butyrate (2.4–2.8 times), its activity severely decreased by 87% *vs.* glucose-cultured cells, which correlated with a lower degree of (Lys) acetylation (data not shown). For HCT 116 cells cultured with acetate (*+*glucose), a significant increment in the AcK content was also observed; however, activity remained unchanged *vs.* glucose-cultured cells, correlating again with a high degree of AcK acetylation (**Supplementary Figure 3A**). Similarly, no correlation between AcK content and activity was found in HCT 116 cells exposed to butyrate or propionate. Propionate or butyrate significantly decreased the AcK activity by 87%. Although its content increased by 1.7 and 3.5 times, respectively (**Figure 3A** and **Table 2**), the AcK acetylation was significantly diminished (data not shown).

**Table 2:** Maximal activity (mU/mg cellular protein) of acetate thiokinase in colorectal cancer cells exposed to short-chain carboxylic acids.

Substrate	COLO 205	HCT 116
Glc	330 ± 181	120 ± 10
Glc + Ac	899 ± 143*	117 ± 20
Glc + Prop	45 ± 18*	22 ± 14*
Glc + But	35 ± 4*	12 ± 2*(3)

Colorectal carcinoma cells were grown in the presence of only glucose (Glc, 5 mM) or glucose+acetate (Ac, 5 mM), propionate (Prop, 0.1 mM), or butyrate (But, 0.1 mM) for 6 days. Afterwards, cells were harvested and washed as described in the Material and Methods section. Data shown are mean ± S.D. from using at least four different preparations. *P < 0.01 vs. Glc.

In order to demonstrate whether butyrate or propionate directly inhibits the AcK activity, both SCCAs were tested on AcK activity from acetate+glucose-exposed CRC cells. The AcK activity from both CRC cells was inhibited by exogenous 0.1 mM propionate (25 and 52.5%) and 0.1 mM butyrate (92.5 and 19%) in COLO 205 and HCT 116, respectively (data not shown).

In order to assess whether AcK was also elevated in human colorectal tumors, analysis of the AcK content as well as acetylation degree was extended to human colorectal cancer biopsies (**Figure 3B**). As control, the AcK content was also determined in colon samples from non-cancerous animals, because no colon samples from healthy persons were available. A higher content of AcK (13.7 times) and acetylation degree (60%) was indeed found in human CRC biopsies *vs.* rat colon tissue, suggesting that a presumably acetylated AcK isoform was highly expressed in colon cancer tissue as previously reported (50).

As internal control for cancer identification, the K-RAS protein was also assessed in the tissue samples. As expected, the K-RAS protein was not detected in the healthy rat colon tissue, whereas its level was remarkable in human CRC biopsies. Interestingly, AcK levels found in human CRC biopsies were similar to those of K-RAS, suggesting a potential role of AcK as metabolic marker of human colon cancer. Analysis of AcK and its acetylation profile and K-RAS protein content was also performed in colon tissue from AS30D hepatoma-bearing animals. AS-30D hepatoma is a fast-growth ascitic cancer model that develops in the rodent peritoneal area close to colorectal tissue. This presumed interaction of AS-30D hepatoma cells with colon tissue for 5–7 days promoted a significant increase of the colon levels of AcK (8.8 times) and to lesser extent of K-RAS *vs.* colon from non-cancer-bearing animals, suggesting a change in the colon metabolism induced by hepatoma cells (**Figure 3B**). However, the high content of AcK in colon tissue from AS-30D hepatoma-bearing animals did not correlate with a high AcK (Lys) acetylation degree, as was observed in human CRC cell lines, suggesting that in the colon of hepatoma rats, some deacetylations may occur, affecting the AcK acetylation profile (**Supplementary Figure 3B**).

### Effect of SCCAs on Cellular ROS Production

**Figure 4:**
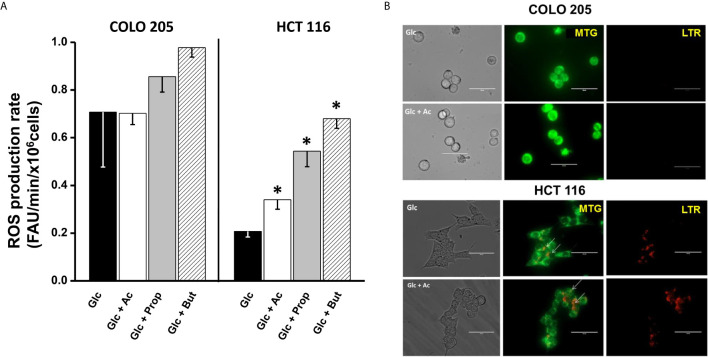
Effect of SCCAs on ROS production **(A)** and mitophagy **(B)** in COLO 205 and HCT116 cells. For ROS production, data shown represent the mean ± S.D. @ of three independent cultures; *P < 0.05 *vs*. Glc. For mitophagy, representative confocal microscopy images of MTG- and LTR-loaded COLO 205 or HCT 116 cells exposed to 5mM glucose (Glc) or 5 mM glucose + 5 mM acetate (Glc + Ac) are shown. Abbreviations as in **Figure 2**.

For ROS detection in acetate-, propionate-, or butyrate-treated cancer cells, the DHE fluorescent probe was used. To verify DHE specificity for ROS detection, the antioxidant cell-permeable N-acetylcysteine (NAC, 2 mM) was used. NAC blocked the DHE signal by >70% (data not shown), indicating that DHE was indeed sensing cell ROS, as previously demonstrated by others (51, 52). Increments in the intracellular ROS levels are frequently associated to OxPhos impairment (53, 54). Thus, the low OxPhos flux detected in HCT 116 cells incubated with glucose and acetate, propionate, or butyrate (**Figure 2A**) correlated with a significant increase (1.6–3.2 times) in ROS levels (**Figure 4A**), as compared to cells exposed to glucose alone. In contrast, COLO 205 cells incubated with acetate (+glucose) showed similar ROS levels to those found in glucose-exposed cells, correlating with a high OxPhos flux (**Figure 2A**). Exposure of COLO 205 cells to propionate or butyrate promoted a slightly higher ROS production (~1.4 times), which in turn correlated with a lower OxPhos capacity (**Figure 2A**). The basal ROS level with glucose alone was three times higher in COLO 205 cells *vs.* HCT 116 cells (**Figure 4A**), but it has been documented that the stationary state ROS content may differ between cancer cell lines (55–57).

### Effect of SCCAs on Mitophagy and Fusion/Fission Mitochondrial Proteins

Mitochondrial digestion was evaluated by assessing (i) the number of lysosomes and their co-localization with mitochondria in LTR/MTG-loaded cells by confocal microscopy (**Figure 4B**, upper panel); and (ii) the content of several autophagy proteins such as PINK-1, PARK, Beclin, DRAM, and LAMP1 (**Supplementary Figure 4A**). For mitophagy detection by confocal microscopy, mitochondrial subcellular localization was revealed with the dye MTG, which covalently binds to the thiols of mitochondrial proteins and accumulates in the mitochondrial matrix regardless of the mitochondrial transmembrane electrical potential. Cells were also loaded with the dye LTR, which accumulates inside lysosomes, autophagosomes, lysosomes, and autolysosomes by virtue of their internal acidic pH.

Confocal images revealed the presence of MTG-loaded mitochondria with apparently high mitochondrial membrane potential in COLO 205 cells incubated with acetate, and the absence of lysosomes. These acetate-incubated cells expressed autophagy proteins (PINK-1 and DRAM) at similar levels to those found in glucose-cultured cells (**Supplementary Figure 4A**). On the other hand, abundant yellow spots (as indicative of co-localization of MTG mitochondria and LTR lysosomes) were detected in HCT 116 cells, revealing an active mitochondrial digestion induced by the presence of acetate (**Figure 4B**, lower panel). The high number of co-loading spots in HCT 116 cells correlated with a significant increment in Beclin and PARK levels, whereas those of DRAM, PINK-1, and LAMP1 remained unchanged (**Supplementary Figure 4A**). For both CRC cells, the presence of propionate or butyrate decreased the number of MTG-loaded mitochondria and increased the number of LTR-loaded lysosomes (data not shown). In parallel, the levels of PINK-1 (1.5–2.7 times), PARK (2.2–5 times), DRAM (1.5–2.3 times), and LAMP1 (3–38 times) significantly increased, indicating mitophagy activation induced by propionate or butyrate.

A second molecular mechanism involved in the regulation of the number of functional mitochondria is related to the mitochondrial fission/fusion processes (58). Indeed, a significant increase (12.5 times) in the fission protein FIS-1 in HCT 116 cells cultured with acetate revealed a significant mitochondrial fragmentation (59) *vs.* COLO 205 cells (**Supplementary Figure 4B**). On the other hand, the protein mitofusin (MNF-1) involved in the mitochondrial fusion, an important process for the maintenance of functional mitochondria (60), was significantly increased in COLO 205 *vs.* HCT 116. These results correlated with the high OxPhos flux found in COLO 205 in the presence of acetate. Propionate or butyrate induced a strong FIS-1 overexpression, similar to that observed for autophagy proteins PINK and PARK, suggesting that these SCCAs may have impaired mitochondrial function through mitophagy and/or mitochondrial fission activation.

### Effect of SCCAs on Colorectal Cancer Cell Invasiveness

**Figure 5:**
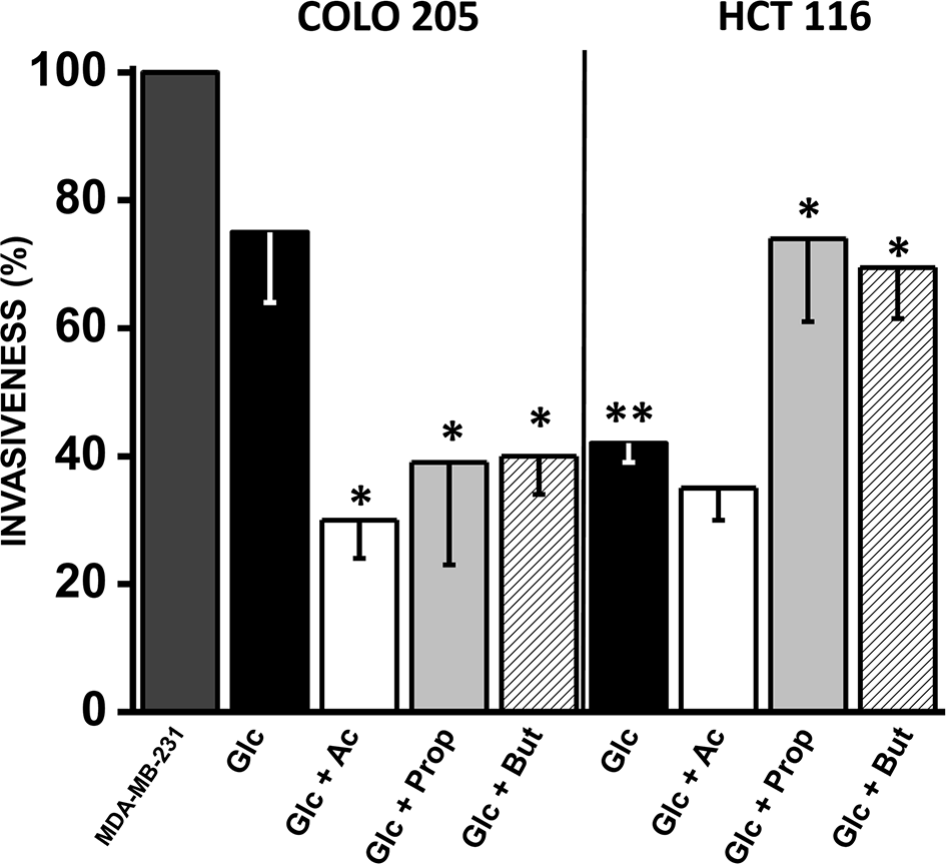
Effect of SCCAs on invasiveness of COLO 205 and HCT 116 cells. The data shown represent the mean ± S.D. @ of at least three different preparations. *P < 0.05; **P < 0.01 *vs.* Glc. Abbreviations as in **Figure 2**.

For invasiveness, metastatic cells require an intensive supply of mitochondrial ATP (61). Therefore, in order to establish whether activation of OxPhos by acetate in COLO 205 cells correlated with the onset and development of the invasiveness process (**Figure 5**), several of the proteins involved as well as the cancer cell invasiveness itself were evaluated (**Supplementary Figure 5**).

A significant change (1.7–4.7 times) in fibronectin (COLO 205 and HCT 116) and vimentin (HCT 116) levels were detected in acetate-exposed cells *vs.* only glucose (**Supplementary Figure 5**). However, no changes were detected in the levels of SNAIL, E-cadherin, and vimentin in acetate-exposed COLO 205 cells. Both CRC cells exposed to butyrate or propionate increased the levels (3–11 times) of several invasiveness proteins, whereas E-cadherin was significantly decreased (56–92%) (**Supplementary Figure 5**).

As positive control for maximal cell invasion (*i.e.*, 100%), the well-known metastatic human breast cancer MDA-MB-231 cells, cultured in 25 mM glucose, were used (**Figure 5**). Cancer cell invasiveness was significantly diminished by acetate, propionate, or butyrate in COLO 205 cells by 30–40% *vs*. cells with only glucose, and 60–70% *vs.* MDA-MB-231 cells. For HCT 116 cells, their invasion profile was similar in acetate (+glucose) and only glucose, and significantly lower *vs.* COLO 205 cells cultured in glucose medium or *vs.* MDA-MB-231 cells. Surprisingly, propionate and butyrate stimulated HCT 116 cellular invasiveness by 75% *vs.* only glucose (**Figure 5**).

## Discussion

### Acetate Increases COLO 205 Cell Proliferation Through AcK and OxPhos Activation

Acetate represents more than 60% of the total SCCAs generated by colon-inhabiting bacterial consortia (62). Then, this simple two-carbon compound may have an important role as fuel as well as signaling molecule, or perhaps as transcriptional cofactor or metabolic modulator, to sustain CRC cell growth (63). Indeed, acetate promoted a significant increase in COLO 205 proliferation (*cf.* **Figure 1A**), like observed in other colorectal (HT-29) and gastric (AGS) carcinomas exposed to acetate (2–6.25 mM) for 3–16 days (64, 65). Proposed mechanisms induced by acetate include (a) associated histone acetylation of genes coding for proteins involved in cellular growth (66); (b) acetylation of non-histone proteins or transcription factors involved in cancer cell proliferation (21, 67); (c) increased levels of the proliferation marker Ki67 (64); and (d) increased mRNA and protein levels of some proinflammatory cytokines and interleukins associated with cell growth activation such as IL-1β, IL-8, and TNF-α (65).

In addition to the above mechanisms, acetate may support COLO 205 cells’ growth by serving as fuel and activating OxPhos for ATP supply. To this end, a dynamic cytosolic/mitochondrial acetate transport/diffusion and an active mitochondrial AcK and functional OxPhos are required. It has been documented that [^14^C] acetate (1 mM) is rapidly transported into HCT-15 CRC cells through overexpressed and active monocarboxylate transporters (MCT1 and/or MCT2), whereas acetate enters RKO cells by facilitated diffusion *via* aquaporins (68). In the cytosol, acetate is actively transformed by a cytosolic acetate thiokinase (AcK), which is also named acetyl-CoA synthetase-2 (ACSS2) (69), in an ATP-dependent reaction to form acetyl-CoA. This activated acetate may be used for fatty acid synthesis (70) that is required for the *de novo* plasma membrane biosynthesis to support cellular growth. An acetate fraction is efficiently transported into mitochondria and transformed by a mitochondrial AcK or ACSS (isoforms 1 and 3) (71), entering the Krebs cycle as acetyl-CoA and activating OxPhos.

The absence of a specific antibody against cytosolic or mitochondrial AcK isoforms precluded the precise identification of the mitochondrial AcK in our study. However, a high mRNA content of mitochondrial AcK has been detected in hepatocarcinoma SNU 449 cells exposed to acetate (72, 73), suggesting that acetate preferentially induces the mitochondrial AcK isoform. Although there are no other studies where mitochondrial (and cytosolic) AcK activity has been experimentally determined in cancer cells, experimental evidence (AcK enzyme activity; AcK protein content and OxPhos flux closely related with the acetate presence) shown in the present work indicated that acetate could be actively oxidized. However, the role of acetate supporting a non-catabolic role in CRC cells cannot be excluded.

Acetate oxidation leads to a high Δψ_m_ for ATP synthesis in hepatoma AS-30D-isolated mitochondria (Hep-M, *c.f.* **Supplementary Table 2**) at comparable rates to those of other canonical mitochondrial fuels such as pyruvate, glutamate/malate, glutamine, or FFAs (23). Acetate promoted (i) a slight uncoupling effect in liver mitochondria but not in hepatoma mitochondria (inserted table in **Figure 2B**) and (ii) elevated citrate levels for NADH production. These observations imply the presence of a highly active mitochondrial AcK, and active acetate metabolism, in cancer mitochondria.

AcK activity in cancer cells has not been previously determined probably because it has been assumed that (i) high AcK mRNA and/or protein contents or (ii) high AcK deacetylation levels induced by Sirt are tightly associated with high enzyme activity (72, 74), which is not always the case (75), and hence activity must be directly determined.

Thus, the direct measurement of COLO 205 AcK revealed that acetate increased its activity as well as its protein level; acetate also increased the HCT 116 AcK protein level but not its activity. These observations suggest that acetate might act as a transcriptional cofactor. The AcK *Vmax* values in COLO 205 and HCT 116 cells cultured with only glucose or glucose+acetate were in the range reported for some microorganisms (76, 77), but they were higher than those reported for mammalian cells (78).

The high AcK activity in COLO 205 cells exposed to glucose+acetate correlated with an enhanced OxPhos, whereas glycolysis remained unchanged. Similar observations were described for human CRC biopsies, where OxPhos increased >50% *vs*. healthy colon tissue, with no apparent change in glycolysis rate (79). COLO 205 cells cultured in glucose medium were also strongly dependent on OxPhos for ATP supply (85%). The addition of acetate promoted that COLO 205 cells depended almost exclusively on OxPhos (>95%). These observations indicated that acetate oxidation prompted OxPhos activation as the principal mechanism for ATP production in COLO 205 cells.

An increased copy number of mitochondrial DNA (mtDNA) was suggested as possible mechanism related with OxPhos activation in patient-derived microsatellite-stable CRC tissue samples (80). Other mechanisms associated to mitochondrial stabilization induced by acetate in CRC were identified, which have not been previously studied in CRC cells: active mitochondrial biogenesis (as judged by the PGC-1α level) and high mitochondrial fusion activity (as judged by the MFN-2 level). An elevated PGC-1α content has also been detected in brown adipocyte IM-BAT cells exposed to acetate (10 mM/7 days) (81). Increased mRNA levels of mitochondrial fusion proteins MFN-1, MFN-2, and OPA-1 were detected in human islets of Langerhans cells exposed to 1 mM acetate for 24 h (82). These acetate-induced mechanisms, along with a higher mtDNA copy number, may operate in COLO 205 cells stimulating OxPhos, increasing cell proliferation and perhaps activating survival mechanisms to avoid apoptosis induction as occurs in human CRC tissue samples (83).

Lysine acetylation has been identified as a widespread post-translational modification of several metabolic enzymes, including mitochondrial proteins (84). In this regard, acetate exposure increased lysine acetylation of mitochondrial AcK in COLO 205 cells correlating with an increment in its activity. In contrast, in HCT 116 cells where AcK activity was significantly lower *vs.* COLO 205, AcK lysine acetylation was also lower. This observation contradicts reports about mice liver and AcK purified enzyme, where AcK acetylation of a specific lysine residue blocks enzyme activity by 40–90% (50, 85, 86).

The differences in the acetylated AcK activity between non-cancer and cancer cells may be attributed to the acetylation of lysine residues directly involved in catalysis of AcK. In this regard, it has been documented that bacterial AcK could be acetylated at four different lysine residues, but only one is responsible of its activity inhibition (87). It has been shown for some glycolytic enzymes such as phosphoglycerate kinase-1 that acetylation of a single particular lysine residue (K220) may block enzyme activity (88), whereas acetylation of another lysine residue (K323) may increase activity (67). The acetylation of either lysine residues depends on the acetylases (PCAF and KAT9) and deacetylases (SIRT7 and HDAC3) expression and activity.

Perhaps, a similar dual response is also implicated in the AcK regulation by acetate. There are no available studies showing the acetylation degree of AcK in cancer cells exposed to acetate. Interestingly, the acetylation level of the AcK from human CRC biopsies was higher than the acetylation of COLO 205 cells AcK, suggesting that AcK protein in biopsies may also maintain a high activity.

It should be noted that aside from acetate, 4 mM glutamine was also present in the CRC culture medium, acting as an essential fuel for cancer proliferation (89). However, glutaminolysis (i.e., lactate production from glutamine breakdown) did not change in both CRC cells under all tested conditions (data not shown), suggesting that the principal carbon source for ATP synthesis, at least in COLO 205 cells, was acetate (+glucose) rather than glutamine.

### Acetate Increased Glycolysis Rate to Sustain HCT 116 Cell Growth

Acetate did not affect growth in HCT 116 cells, in agreement with data previously reported (90). Apparently, acetate does not modify the histone acetylation profile, and consequently cell growth was not stimulated.

Energy metabolism in HCT 116 cells was completely different than that of COLO 205 cells. HCT 116 cells maintained a predominant glycolytic metabolism (high HIF-1α and glycolytic protein contents) in glucose or glucose + acetate medium, with OxPhos playing a minor role. There are no available studies examining the effect of acetate on glycolysis flux in cancer cells; however, it has been found that acetate increases the HIF-1α and HIF-2 levels in human fibrosarcoma HT1080, but after prolonged hypoxia (1% O_2_) (91). This observation suggests a potential transcriptional role for acetate.

None of the assayed SCCAs, including acetate, modified the OxPhos flux in HCT 116 cells. In addition, the OxPhos flux of HCT 116 cells was similar to that previously reported (92), but it was significantly lower than that of COLO 205 cells and other cancer cells studied previously like MCF-7, MDA-MB-231, MDA-MB-468, and HeLa cells (48, 61). The low OxPhos capacity of HCT 116 cells correlated with their low total AcK protein activity (*c.f*. **Table 2**) as well as with an active mitochondrial digestion (*c.f*. **Figure 4B**), indicating that HCT 116 cells have an impaired mitochondrial function.

Contrary to what was observed for AcK, most acetate-induced OxPhos or glycolysis enzymes were not acetylated by exogenous acetate, except 2-OGDH, GLUT-1, and HKII. It has been documented for non-cancer cells that acetylation of some mitochondrial enzymes such as ICDH, respiratory chain Complex I, GDH, or MDH promotes activity inhibition. However, in other enzymes such as enoyl-CoA hydratase or aconitase, acetylation increases activity [reviewed in (93)]. Unfortunately, there are no reports about the effects of acetylation on these energy-metabolism proteins and its correlation with their activity and stability in cancer cells.

It is important to emphasize that clear metabolic differences were found in both COLO 205 and HCT 116 cells after acetate exposure. These differences may be attributed to the following: (1) Different expression and activity of acetylase and deacetylase isoforms found in COLO 205 and HCT 116 cells (94). For instance, COLO 205 cells show a histone deacetylase activity 20% lower than that found in HCT 116 cells; and gene expression of particular deacetylase isoforms (i.e., HDAC 1-5) is different between COLO 205 and HCT 116. These events, among others, promote different epigenetic modifications affecting a wide range of cellular functions (95). (2) Remarkable phenotypic differences between COLO 205 and HCT 116 cells are apparent when they are cultured. COLO 205 cells were derived from the ascites region*, i.e*., these CRC cells underwent metastasis, which may explain why they show high invasion capacity (*cf.* **Figure 5**), whereas HCT 116 cells were derived from a primary colorectal solid tumor, whose invasion profile is significantly lower (96, 97). (3) Differences in the mutated or non-mutated (wild-type) status of transcription factors, such as p53, might promote differences in cellular responses (98, 99). In this regard, it has been documented that HCT 116 cells express wild-type p53, whereas COLO 205 contain a mutated p53 isoform, which most likely affects energy metabolism fluxes, drug sensitivity (99, 100), and most likely some other cell functions. This last observation may explain the observation that HCT 116 cells are acetate-producing cancer cells (101), instead of acetate-consuming cells like COLO 205.

### Molecular Mechanisms Associated to the Impairment of Proliferation and OxPhos by Propionate or Butyrate in CRC Cells

Contrary to normal colonocytes (102, 103), physiological butyrate or propionate concentrations decrease CRC cell proliferation (*c.f.* **Figure 1**). The proposed molecular mechanisms associated to this effect are (1) strong histone deacetylases (HDACs) inhibition, which allows cyclin-dependent kinase inhibitory protein p21/Cip1 transcription, resulting in cell cycle arrest in the G1 phase (90, 104); (2) apoptosis induction through hyperactivation of the WNT/beta-catenin signaling pathway (105); (3) mitophagy induction (present study), increasing the levels of PARK, DRAM, and LAMP1 (*c.f.* **Supplementary Figure 4**) [it has been demonstrated in human CRC HCT 116 and SW480 and human hepatocellular carcinoma Huh 7 cells that mitophagy activation induced by butyrate or propionate occurs through downregulation of its negative regulator mTOR, which in turn promotes AMPKα hyperphosphorylation (106, 107); however, the levels of P-AMPK were unchanged when CRC cells were exposed to butyrate or propionate (**Supplementary Figure 1**)]; (4) active mitochondrial fission (*c.f.* **Supplementary Figure 5**), which, together with mitophagy, decreases the mitochondrial mass and in consequence OxPhos; (5) the direct inhibition of mitochondrial AcK by butyrate and propionate and in consequence the OxPhos flux.

### Invasiveness Processes Are Strongly Affected by SCCAs in CRC Cancer Cells

There are no reports about the effect of acetate on CRC cell metastasis and invasion, whereas the effect of butyrate on cancer cell invasiveness has been widely analyzed. Butyrate at physiological concentrations (0.5–5 mM) increases the expression of metalloproteinases and upregulates miR-3935, which in turn inhibits migration and invasion in HT1080 fibrosarcoma and human A549 lung cancer cells (108, 109). It has been shown in HT29, LOVO, and HCT8 cells that blocking of migration induced by butyrate includes HDAC3, Akt1, and ERK1/2 inactivation (110). In agreement with the latter observations, the three SCCAs inhibited invasiveness of COLO 205 cells, although, in marked contrast, butyrate and propionate stimulated invasiveness of HCT 116 cells. This puzzling result clearly deserves further investigation.

### AcK Overexpression in COLO 205 Cells and Human CRC Biopsies: A Potential Metabolic Marker for Cancer Identification

Human CRC biopsies showed a high AcK level, which was similar to that detected in COLO 205 cells. Interestingly, high levels of AcK and K-RAS were also detected in the colon derived from AS-30D ascites hepatoma-bearing animals (*vs.* colon from healthy rats; *c.f.* **Figure 3B**). This last result clearly indicated that cancer cells may induce changes in neighboring healthy tissues to acquire a metastatic phenotype.

Overexpression of AcK has been found in several CRC cells (111–113) as well as in CRC human biopsies (63, 114). In bladder urothelial BLCA and GCa carcinomas, mitochondrial AcK has been proposed as an enzyme with potential oncogenic role. Thus, it could be used as prognostic biomarker providing a promising novel target for cancer treatment (71, 115). However, it has also been described that inflammatory processes in the bowel, associated to a modified luminal bacterial metabolite composition (116), also increase the AcK protein level (63). Thus, AcK protein detection should be accompanied by other well-known CRC oncogenes such as H-RAS (c.f. **Figure 3B**) or K-RAS (117).

In the present study, animal experimental systems were used instead of human systems under the assumption that some cause-effect relationships are similar in humans and experimental systems. Accordingly, several studies have shown a high similitude in metabolic responses between animal models and human tissue (118, 119). Then, data derived from cultured cancer cells, isolated rat mitochondria, and human biopsies show one common important finding, which may be envisioned as potential alternative to deter CRC growth. (1) Some CRC cells acquire, *via* expression of mitochondrial AcK, a high acetate dependency for their growth. Elevated AcK levels were also found in cancer biopsies and cancer-isolated mitochondria. Then, selective inhibitors of this enzyme represent an opportunity to develop new anticancer therapeutics. In this regard, over 100,000 molecules capable of selectively inhibiting AcK have been recently discovered (120). As AcK is also found in healthy tissues like colon, chemotherapy should be oriented to use multi-target drugs for increasing potency and selectivity and decreasing adverse side-effects. (2) The present data suggest that acetate-producing bacteria (*i.e.*, enteric bacteria) in the gut of healthy patients may favor the development of CRC, whereas propionate- and butyrate-producing gut bacteria may disfavor CRC growth. Therefore, the identification of a metabolic diet that may modulate the gut microbiome may be useful to decrease CRC growth.

## Data Availability Statement

The original contributions presented in the study are included in the article/**Supplementary Material**. Further inquiries can be directed to the corresponding authors.

## Ethics Statement

The studies involving human participants were reviewed and approved by Committees of Ethics and Research of Instituto Nacional de Cancerología, México (INCAN) (http://incan-mexico.org/incan//pub/investigacion/bioetica/Anexo7.pdf). The patients/participants provided their written informed consent to participate in this study. The experimental protocols for the use and care of laboratory animals followed the guidelines of the Norma Oficial Mexicana (NOM-062-ZOO-1999) and for disposal of biological residues (NOM-087-SEMARNAT-SSA1-2002).

## Author Contributions 

SR-E and RM-S: conception and design; analysis; interpretation of data; study supervision; manuscript writing; and manuscript revision. DR-C, JG-P, SP-V, CV, JV-N, BB-C, and RE: development of methodology and manuscript proofreading. ES, ÁM-H, RJ-C, LR-G, and JA-P: analysis, interpretation of data, and revision of manuscript. All authors contributed to the article and approved the submitted version.

## Funding

This work was partially supported by CONACyT-México grant Nos. 283144 (SR-E), 377873 (SP-V), 282663 (ES), and 6379 (RM-S). 

## Conflict of Interest

The authors declare that the research was conducted in the absence of any commercial or financial relationships that could be construed as a potential conflict of interest.

## Publisher’s Note

All claims expressed in this article are solely those of the authors and do not necessarily represent those of their affiliated organizations, or those of the publisher, the editors and the reviewers. Any product that may be evaluated in this article, or claim that may be made by its manufacturer, is not guaranteed or endorsed by the publisher.

## Abbreviations

Ac, acetate; AcK, acetate thiokinase; ANT, adenine nucleotide translocase; ATPS, ATP synthase; CRC, colorectal cancer; But, butyrate; DHE, dihydroethidium; DRAM, damage-regulated autophagy modulator; FAU, fluorescence arbitrary units; FIS-1, mitochondrial fission-1 protein; Glc, glucose; Glu, glutamate; GLUT-1, glucose transporter 1; GLUT-3, glucose transporter 3; Hep-M, rat AS-30D hepatoma mitochondria; HKI, hexokinase I; HKII, hexokinase II; K-RAS, Kirsten rat sarcoma viral oncogene homolog; LAMP1, lysosomal associated-membrane protein-1; Mal, malate; MFN-2 mitofusin-2; ND1, NADH dehydrogenase subunit 1; OxPhos, oxidative phosphorylation; P-AMPK, phosphorylated AMP-activated protein kinase; PARK, parkin; PGC-1α, peroxisome proliferator activated receptor-gamma coactivator-1 alpha; PINK-1, PTEN induced-kinase 1; Prop, propionate; Pyr, pyruvate; RLM, rat liver mitochondria; ROS, reactive oxygen species; SCCAs, short-chain carboxylic acids.
