# Supplementary material for: Acetate Promotes a Differential Energy Metabolic Response in Human HCT 116 and COLO 205 Colon Cancer Cells Impacting Cancer Cell Growth and Invasiveness
Source: Front Oncol. 2021 Aug 3;11:697408. doi: 10.3389/fonc.2021.697408 (PMC8370060; doi:10.3389/fonc.2021.697408)
Supplement: Supplementary file 1 [file DataSheet_1.pdf]

SUPPLEMENTARY MATERIAL

Acetate promotes a differential energy metabolic response in human HCT 116 and COLO 205 colon cancer cells impacting cancer cell growth

Sara Rodríguez-Enríquez[1,*], Diana Xochiquetzal Robledo-Cadena[1], Juan Carlos Gallardo-Pérez[1], Silvia Cecilia Pacheco-Velázquez[1], Citlali Vázquez[1], Emma Saavedra[1], Jorge Luis Vargas-Navarro[1], Betsy Alejandra Blanco-Carpintero[1], Álvaro Marín-Hernández[1], Ricardo Jasso-Chávez[1], Rusely Encalada[1], Luz Ruiz-Godoy[2], José Luis Aguilar-Ponce[3] and Rafael Moreno-Sánchez[1,*]

1. Departamento de Bioquímica, Instituto Nacional de Cardiología, México, MEX.
2. Banco de Tumores, Instituto Nacional de Cancerología, México, MEX.
3. Departamento de Medicina Interna, Instituto Nacional de Cancerología, México, MEX.

*Corresponding authors:

Sara Rodríguez-Enríquez, Ph. D.
Email: sara.rodriguez@cardiologia.org.mx; saren960104@hotmail.com.
Rafael Moreno-Sánchez, Ph. D.
Email: rafael.moreno@cardiologia.org.mx

Supplementary Table 1

SCCAs oxidation in rat liver (RLM) isolated mitochondria

RLM	Pseudo state 4 respiration (no ADP added)	State 3 (ADP-stimulated) respiration	State 4 respiration (after ADP exhaustion)	Respiratory control ratio
No added Substrates	60±22	60±22	60±22	1
1 mM Pyr + Mal	36±8	77±28	41±35	2±1
5 mM Glu + Mal	51±15	209± 61	26±12	8± 1
Ac (mM) + Mal				
1	54±27	73±33	31±11	3±1
5	49±19	99±18	66±40	2±1
10	38±11	73±32	53±48	2±1
Prop (mM) + Mal				
1	36±5	53± 16	29 ± 17	2 ± 1
5	32± 8	64 ± 14	46 ±38	2 ± 1
10	41 ± 12	65 ± 15	46 ±41	2±1
But (mM) + Mal				
1	50±31	100±22	60±49	2±1
5	34±14	93±35	30±15	2±1
10	31±14	75±16	50±42	2±1

Mal was used at a concentration of 5mM. Data shows the mean ± S.D. of at least 3 independent preparations.

Supplementary Table 2

Effect of SCCAs on mitochondrial transmembrane potential (ΔΨm, FAU) in AS-30D hepatoma (Hep-M) and rat liver (RLM) isolated mitochondria

	Hep-M		RLM	
Added substrate	Pseudo state 4 ΔΨm (no ADP added)	State 3 (ADP-stimulated) ΔΨm	Pseudo state 4 ΔΨm (no ADP added)	State 3 (ADP-stimulated) ΔΨm
1mM Pyr + Mal	132 ± 21 (3)*	135 ± 26(3)	192 ± 25(3)	189 ± 29(3)
5mM Glut + Mal	102 ± 12(3)	100 ± 22 (3)	119 ± 28 (3)	126 ± 29 (3)
5 mM Ac + Mal	184 ± 10 (3)*	179 ± 9 (3) *	154 ± 14 (3)	147 ± 15(3)
5 mM But + Mal	115 ± 29(3)	133 ± 37 (3)	167 ± 52(3)	163 ±45 (3)

Mitochondrial transmembrane electrical potential (ΔΨm) was measured as described in the Material and Methods section. Data shows the mean ± S.D. of at least 3 independent preparations.

Abbreviations: Ac, acetate; But, butyrate; FAU, fluorescence arbitrary units; Glut, glutamate; Mal, malate; Pyr, pyruvate. Added Mal concentration was 0.1 mM for Hep-M and 5 mM for RLM.

Supplementary Table 3

Effect of SCCAs on mitochondrial citrate content in AS-30D hepatoma (Hep-M) and rat liver (RLM) isolated mitochondria

	Hep-M	RLM
	Citrate content (nmol/mg of protein)	
Added substrate		
10 mM Glut + Mal	4.2 ± 1 (3)	1.3 ± 0.2 (3)
5 mM Ac + Mal	5.8 ± 1.5 (3)	2.4 ± 0.7 (3)

Intramitochondrial citrate content was measured as described in the Material and Methods section. Data shows the mean ± S.D. of at least 3 independent preparations.

Abbreviations: Ac, acetate; Glut, glutamate; Mal, malate; Pyr, pyruvate. Added Mal concentration was 5 mM for Hep-M and RLM.

Supplementary figures

Supplementary Figure 1

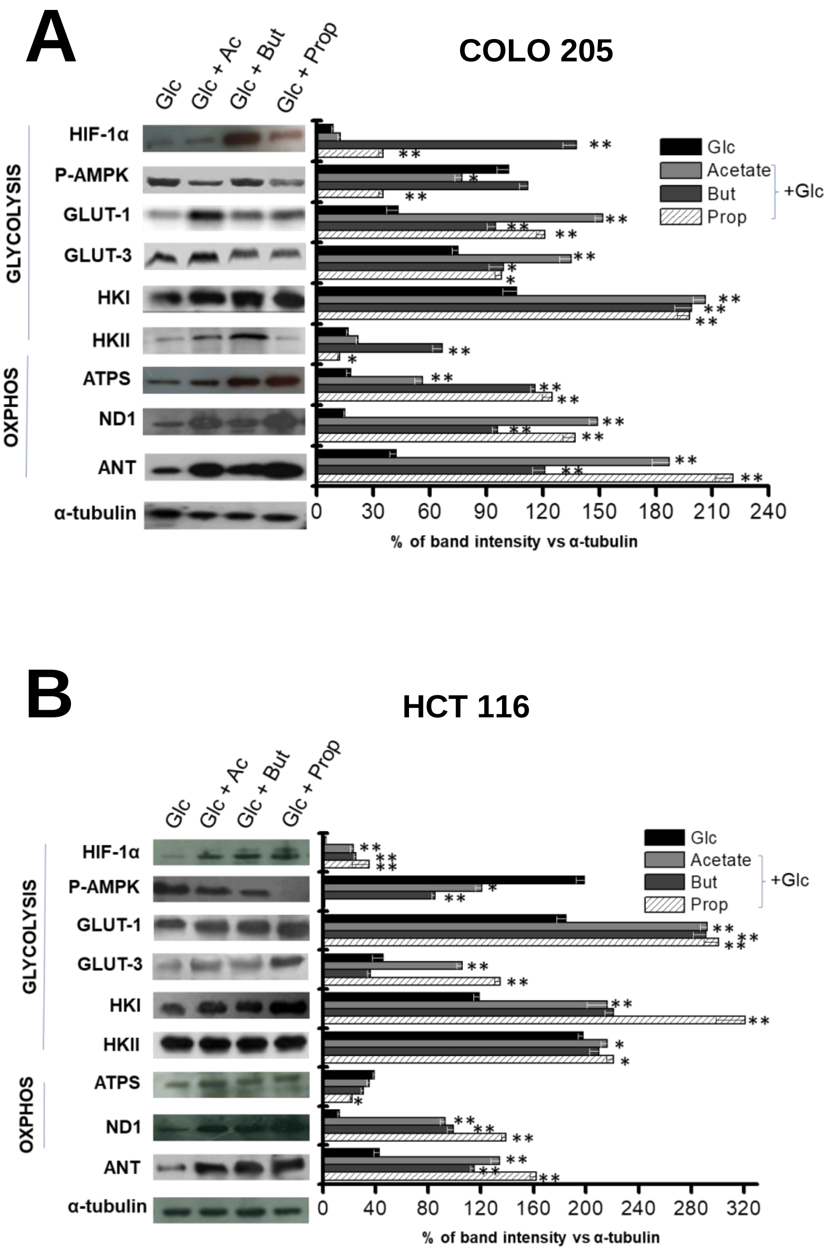

Supplementary Figure 1. Effect of SCCAs on the content of energy metabolism proteins in COLO 205 **(A)** and HCT 116 **(B)** cells. Cells were cultured with the indicated carbon sources (5 mM glucose-Glc; 5 mM acetate-Ac; 0.1 mM propionate-Prop; 0.1 mM butyrate-But) for 5 days, harvested, washed and used for western blotting. The data shown represent the mean ± S.D. of at least three independent preparations.* $P < 0.05$;** $P < 0.01$ vs. Glc**.**

Supplementary Figure 2

A COLO 205

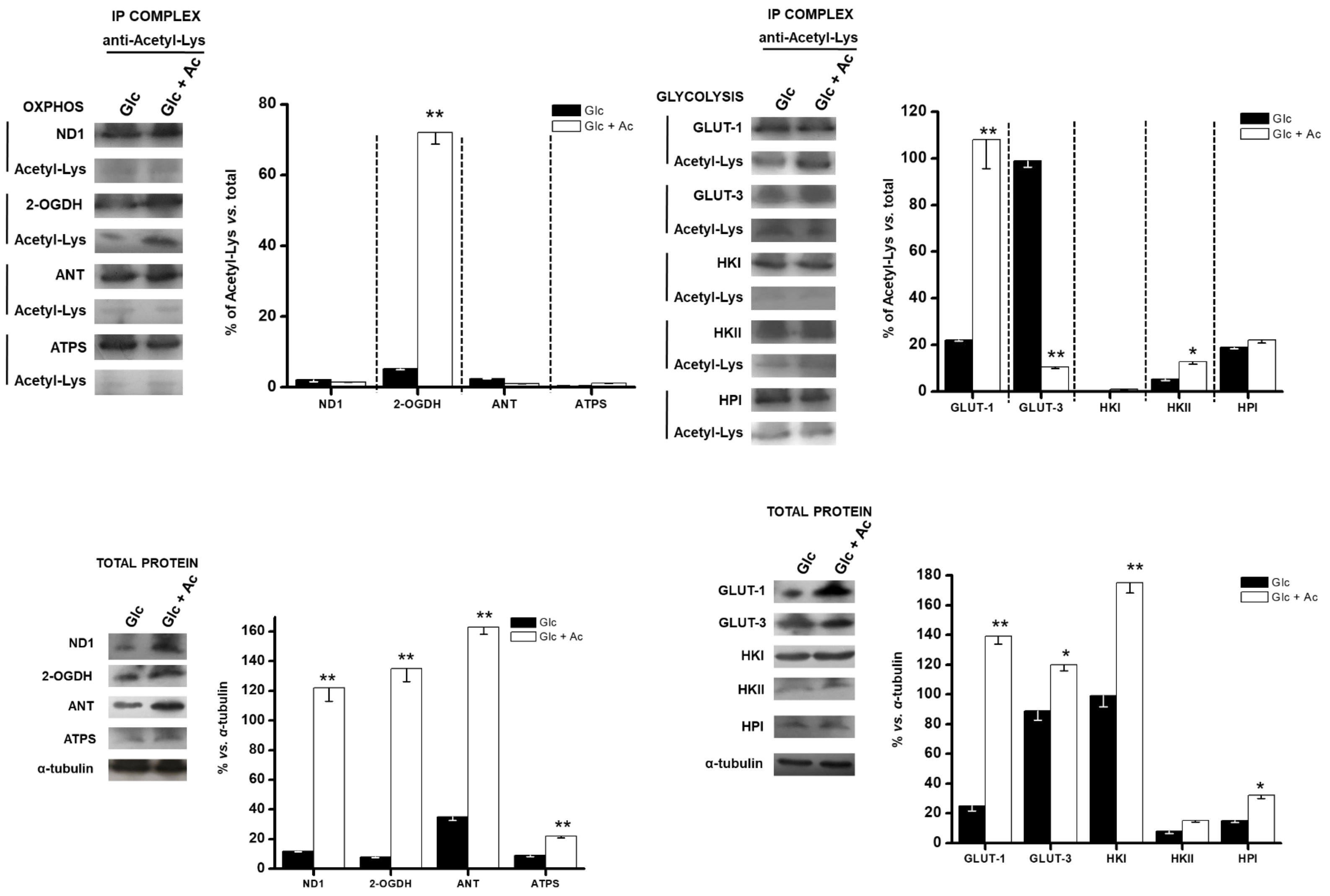

B **HCT 116**

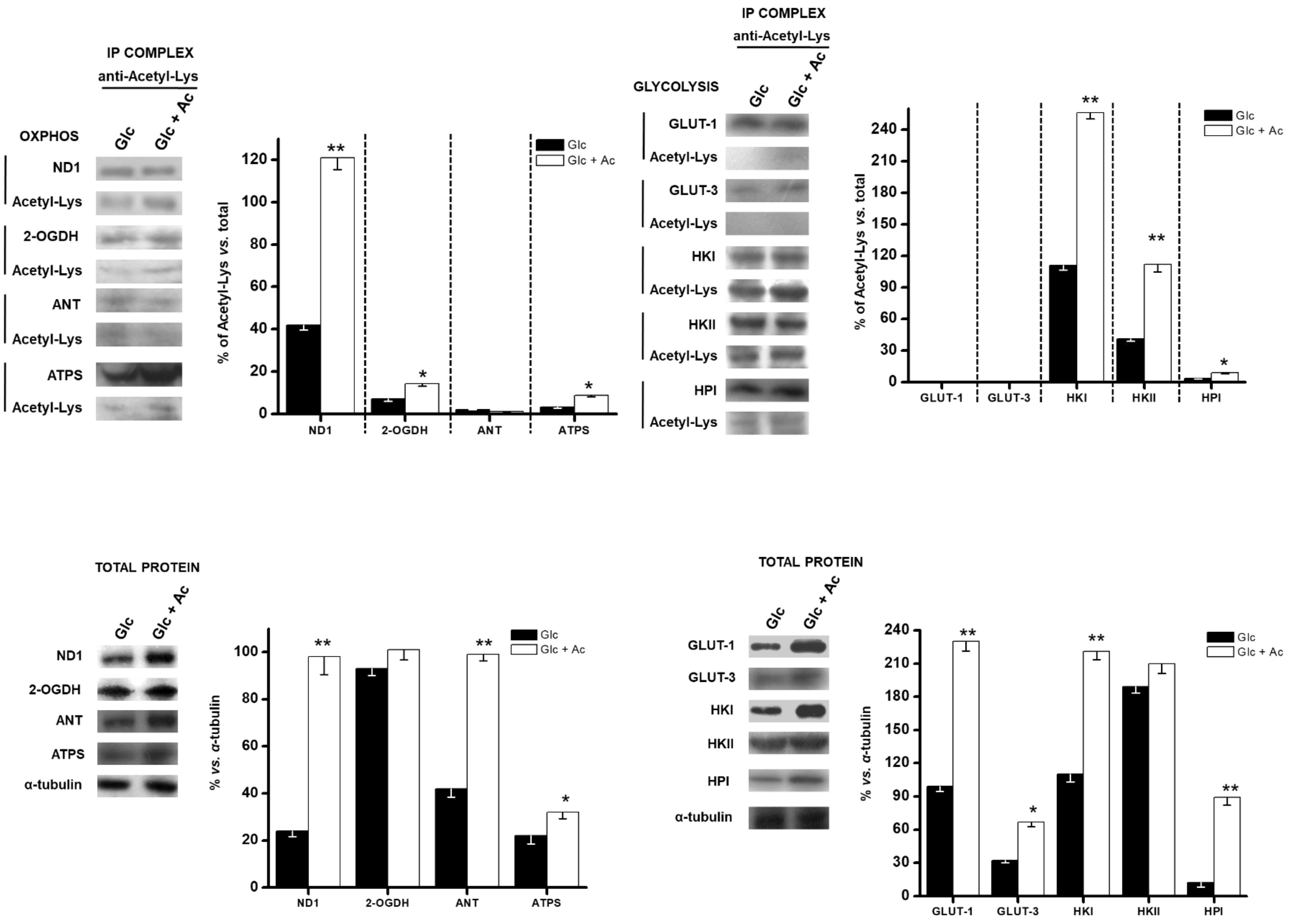

Supplementary Figure 2. Effect of acetate on acetylation of OxPhos and glycolysis proteins in **(A)** COLO 205 and **(B)** HCT 116 cells. Immunoprecipitation (IP, upper panel) and total protein (lower panel) is shown. The data shown represent the mean ± S.D. of at least three different preparations.* P < 0.05;** P < 0.01 vs. Glc. Abbreviations as in **Supplementary Figure 1.**

Supplementary Figure 3

A

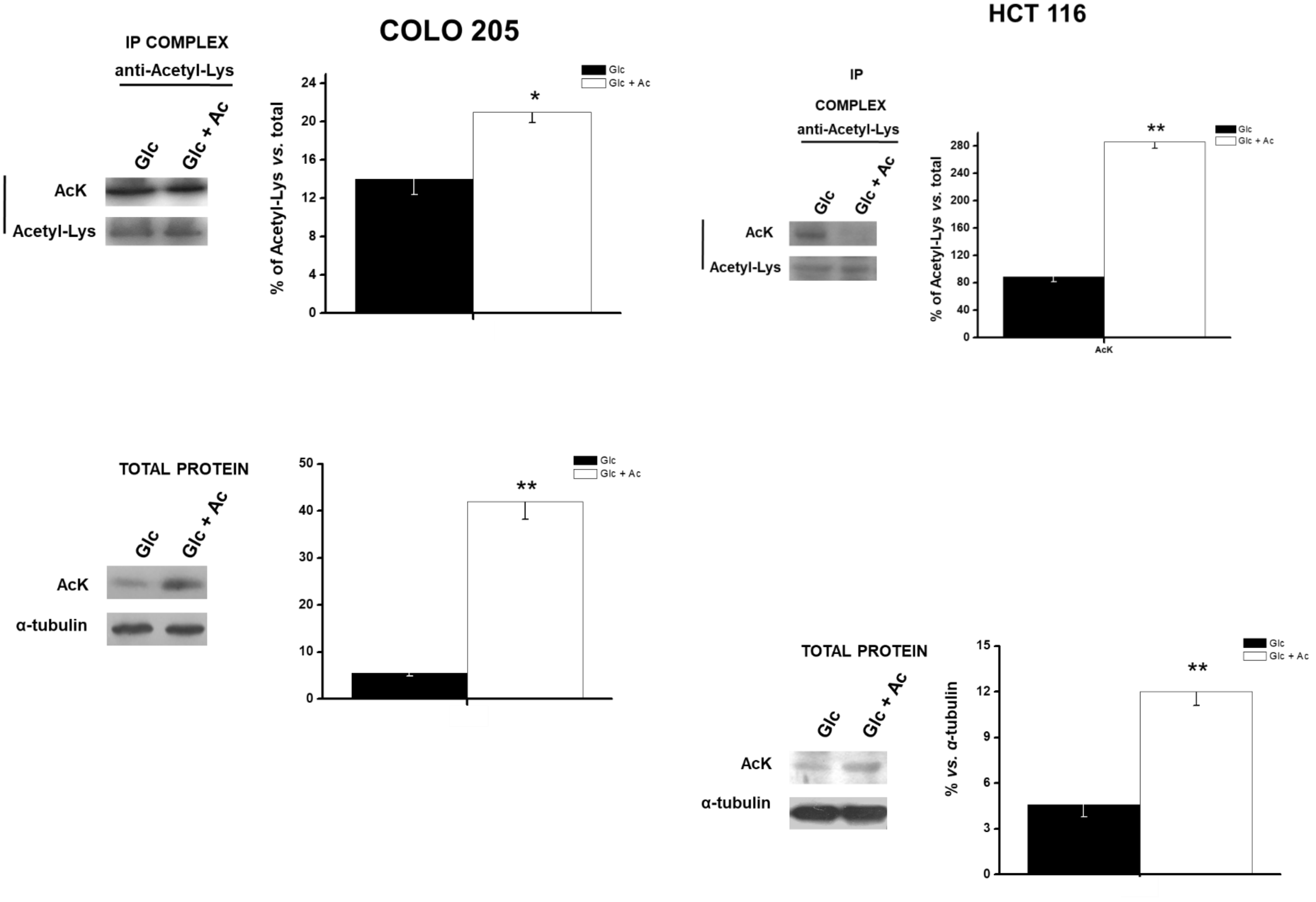

B

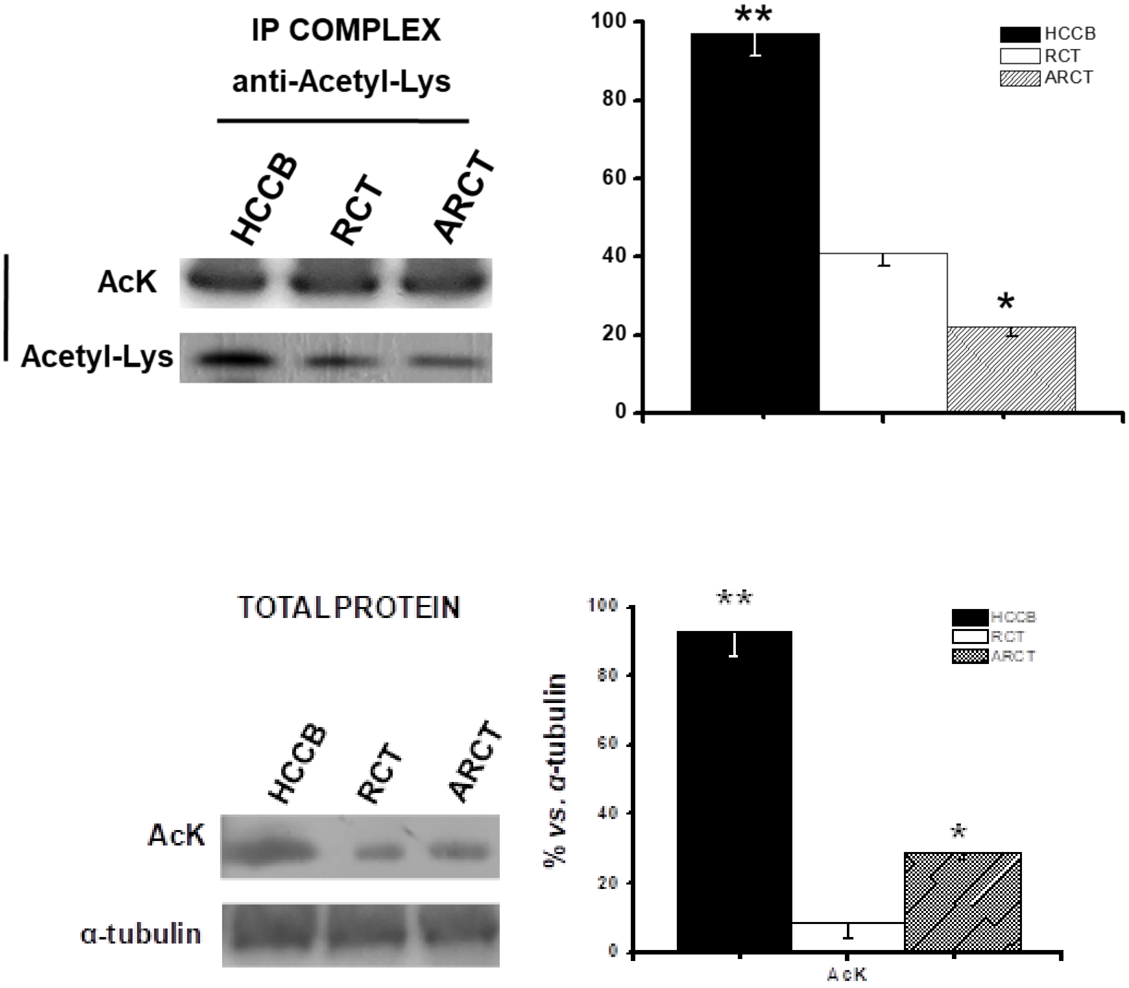

Supplementary Figure 3. (A) Effect of acetate on AcK acetylation in COLO 205 and HCT 116 cells. Immunoprecipitation (IP, upper panel) and total protein (lower panel) are shown. The data shown represent the mean ± S.D. of at least three different preparations.* P < 0.05;** P < 0.01 vs. Glc. Abbreviations as in **Supplementary Figure 1**.

(B) Effect of acetate on AcK acetylation degree in human CRC biopsies and rat colon. Immunoprecipitation (IP) and total protein are shown**.** * P<0.05 vs. rat colon tissue (RCT); ** P<0.01 vs. rat colon tissue (RCT). Abbreviations: HCCB, human colon cancer biopsy; ARCT, ascites rat colon tissue.

Supplementary Figure 4

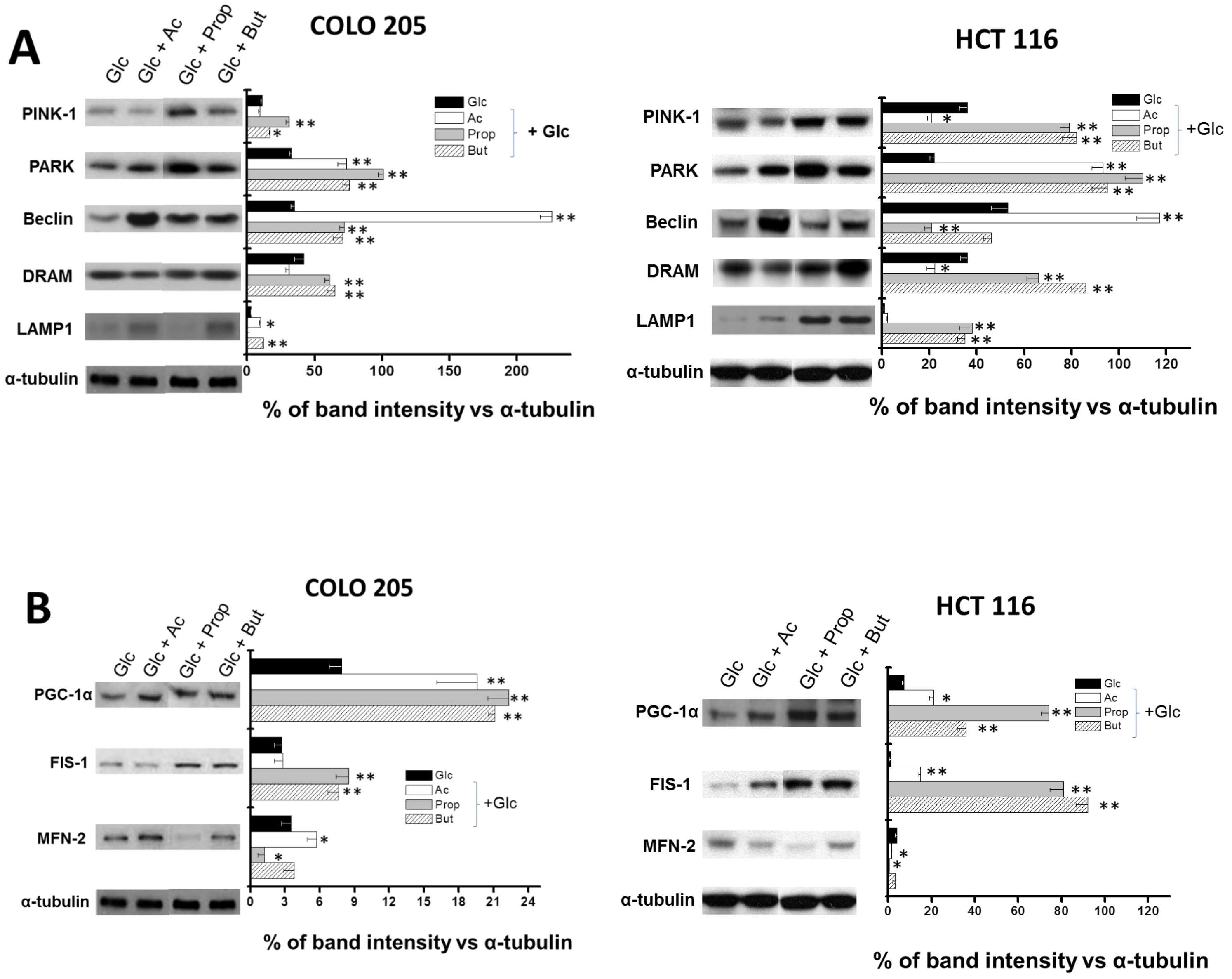

Supplementary Figure 4. Effect of SCCAs on **(A)** mitophagy and **(B)** fission/fusion protein contents in COLO 205 and HCT 116 cells. The data shown represent the mean ± S.D. of at least three different preparations. * P < 0.05;** P < 0.01 vs. Glc. Abbreviations as in **Supplementary Figure 1.**

Supplementary Figure 5

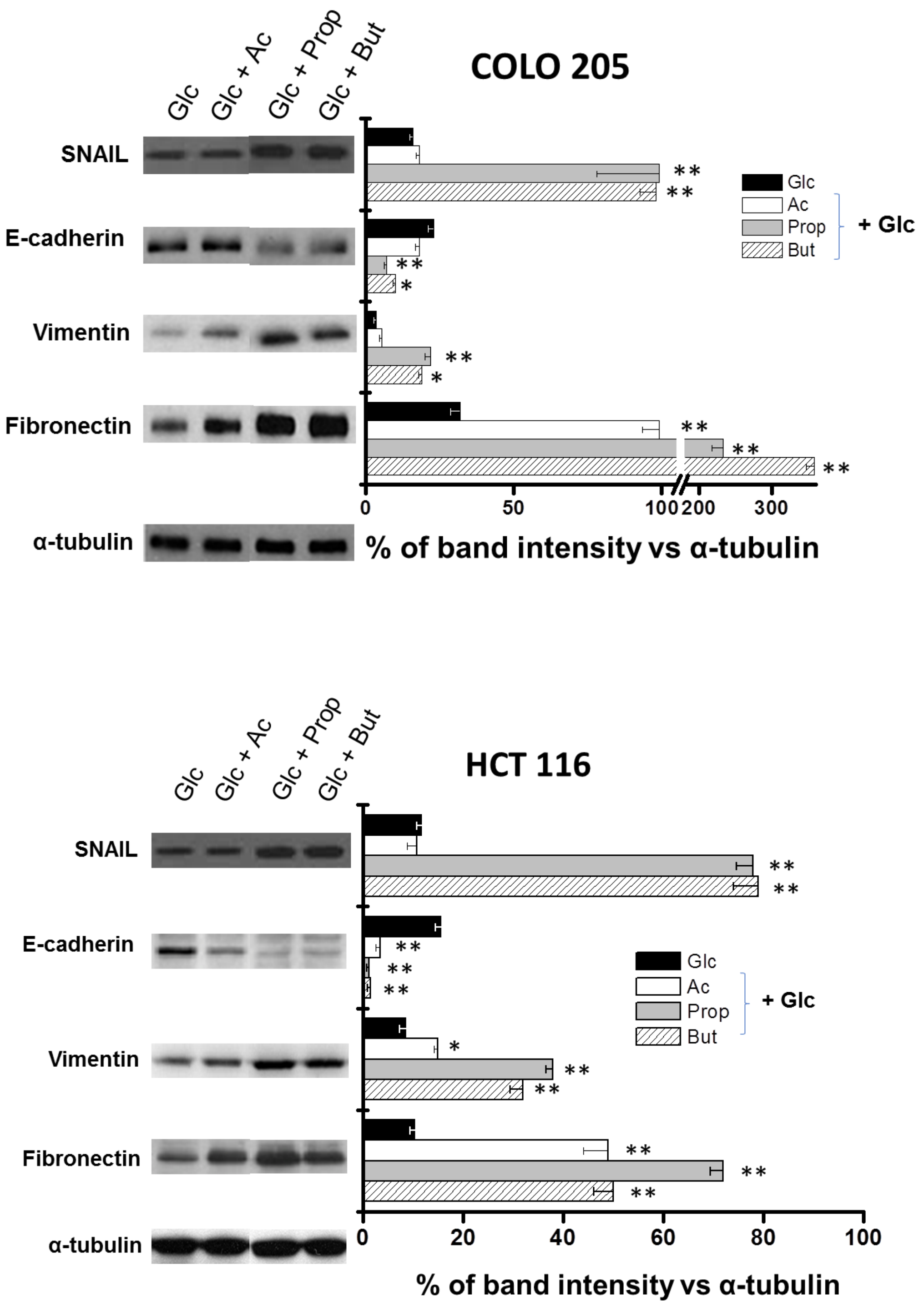

Supplementary Figure 5. Effect of SCCAs on invasiveness protein contents in COLO 205 and HCT 116 cells. The data shown represent the mean ± S.D. of at least three different preparations.* $P < 0.05$;** $P < 0.01$ vs. Glc. Abbreviations as in **Supplementary Figure 1.**
